# Targeting hypoxia regulated sodium driven bicarbonate transporters reduces triple negative breast cancer metastasis

**DOI:** 10.1016/j.neo.2022.01.003

**Published:** 2022-02-09

**Authors:** Christopher Paul Carroll, Hannah Bolland, Eric Vancauwenberghe, Pamela Collier, Alison A. Ritchie, Philip A. Clarke, Anna M. Grabowska, Adrian L Harris, Alan McIntyre

**Affiliations:** 1 Hypoxia and Acidosis Group, Nottingham Breast Cancer Research Centre, School of Medicine, Biodiscovery Institute, University of Nottingham; 2 *Ex Vivo* Cancer Pharmacology Centre, Biodiscovery Institute, University of Nottingham; 3 Molecular Oncology Laboratories, Department of Oncology, University of Oxford, Weatherall Institute of Molecular Medicine, Oxford, United Kingdom

**Keywords:** Hypoxia, Metastasis, Triple Negative Breast Cancer, EMT, NDBT, Tumour Acidosis

## Abstract

Regions of low oxygen (hypoxia) are found in >50% of breast tumours, most frequently in the more aggressive triple negative breast cancer subtype (TNBC). Metastasis is the cause of 90% of breast cancer patient deaths. Regions of tumour hypoxia tend to be more acidic and both hypoxia and acidosis increase tumour metastasis. In line with this the metastatic process is dependent on pH regulatory mechanisms. We and others have previously identified increased hypoxic expression of Na^+^ driven bicarbonate transporters (NDBTs) as a major mechanism of tumour pH regulation. Hypoxia induced the expression of NDBTs in TNBC, most frequently *SLC4A4* and *SLC4A5*. NDBT inhibition (S0859) and shRNA knockdown suppressed migration (40% reduction) and invasion (70% reduction) *in vitro*. Tumour xenograft metastasis *in vivo* was significantly reduced by NDBT knockdown. To investigate the mechanism by which NDBTs support metastasis, we investigated their role in regulation of phospho-signalling, epithelial-to-mesenchymal transition (EMT) and metabolism. NDBT knockdown resulted in an attenuation in hypoxic phospho-signalling activation; most notably LYN (Y397) reduced by 75%, and LCK (Y394) by 72%. The metastatic process is associated with EMT. We showed that NDBT knockdown inhibited EMT, modulating the expression of key EMT transcription factors and ablating the expression of vimentin whilst increasing the expression of E-cadherin. NDBT knockdown also altered metabolic activity reducing overall ATP and extracellular lactate levels. These results demonstrate that targeting hypoxia-induced NDBT can be used as an approach to modulate phospho-signalling, EMT, and metabolic activity and reduce tumour migration, invasion, and metastasis *in vivo*.

## Introduction

More than half of breast tumours contain regions of low oxygen (hypoxia) [1], arising from high metabolic and proliferative rates and aberrant tumour vascularisation [2]. Normal breast median pO_2_ is 65 mmHg, compared with a median pO_2_ of 10 mmHg in breast tumours with regions <2.5 mmHg [3]. Hypoxia results in the stabilization of the hypoxia inducible factor (HIF) transcription factors which induce significant transcriptional changes; upregulating genes that modulate the major hallmarks of cancer including metabolism, metastasis and pH regulation [4]. Clinically, hypoxia is associated with resistance to radiotherapy and chemotherapy [5]. Acidosis is also a common feature of the tumour microenvironment locoregionally associated with hypoxia [6]. Acidosis arises due to increased metabolic activity and reduced ion venting due to poor vascularisation, resulting in a build-up of H^+^ [6]. Breast cancers have a significantly more acidic extracellular pH (pH_e_)(as low as pH 6.5) compared to surrounding tissue (pH 7.2) [7] and a more acidic pH_e_ correlates with a worse patient prognosis and therapy response [8].

The main extracellular and intracellular buffer in human tissue is bicarbonate which regulates pH via interconversion with carbon dioxide [9]. Carbonic anhydrases, CA9 and CA12, regulate pH_e_ in hypoxic tumours by hydrating CO_2_ into bicarbonate and H^+^. CA9 and CA12 are significantly upregulated in hypoxia in a HIF1α dependant manner [10]. CA9 works in conjunction with Na^+^-driven bicarbonate transporters (NDBTs) to regulate intracellular pH (pH_i_) and loss of NDBTs result in lower enzymatic efficiency of CA9 [11]. NDBTs co-transport Na^+^ and HCO_3_^−^ into the cell. There are multiple members of the NDBT family including SLC4A4, SLC4A5, SLC4A7, and SLC4A9. The most studied and highest expressed in breast cancer is SLC4A7 [12]. We and others have previously shown increased hypoxic expression of NDBT, in a HIF1 dependant manner [13,14], in multiple tumour settings (including glioblastoma, and breast and colon cancer). Furthermore, we previously identified that disruption of NDBTs increases apoptosis in the hypoxic core of tumours and delays 3D tumour growth *in vitro* and *in vivo* through regulation of tumour pH_i_ (identified in 2D and 3D culture investigating pH at steady state and using ammonium prepulse) [12,[14], [15], [16]].

Metastasis is the cause of 90% of breast cancer deaths and regions of hypoxia and acidosis correlate with increased occurrence of metastasis [17]. Hypoxia and HIF regulate the expression of genes involved at each stage of the metastatic process, including, genes that increase epithelial mesenchymal transition (EMT), and modulators of the extracellular matrix, Lysyl oxidase (LOX), and matrix metalloproteinases (MMPs) [18]. pH regulatory enzymes induced by HIF ensure a slightly alkaline pH_i_ whilst acidifying the extracellular space. This acidic extracellular pH alters extracellular matrix (ECM) composition, and activates MMPs [19], [20], [21]. In line with this CA9 has a key role in regulation of metastasis [22]. pH regulatory mechanisms prevent accumulation of intracellular H^+^. H^+^ ionise histidine residues altering protein stability, which perturbs signal transduction via key tyrosine kinases [23]. For example intracellular acidosis inhibits mTOR activation and reduces HIF stabilization [24]. The breast cancer subtype triple negative breast cancer (TNBC) is more frequently hypoxic and has a higher incidence of metastasis than other breast cancer subtypes and thus is the focus of our study [13]. Developing targeted therapies to prevent hypoxia and acidosis induced metastasis could be utilised to improve overall patient survival.

Here, we investigate the role of NDBT in metastasis and the mechanistic processes that support this including EMT, phospho-signalling and metabolism. We examine the role of NDBTs using shRNA knockdown and inhibition using S0859. S0859 is an inhibitor of NDBTs which has been identified to have some off-target effects including inhibition of the lactate transporters at concentrations similar to those used in this study which have previously been used to investigate NDBT [12,16,25,26]. We identified increased expression of NDBT in response to hypoxia in TNBC and investigate the role of NDBTs in migration and invasion *in vitro.* We identify a critical role for NDBTs in steady-state pH_i_ regulation, kinase signalling, EMT induction, and metabolic adaption. Finally, we show that NDBTs regulate metastasis *in vivo*. This study demonstrates the importance of NDBTs in pH homeostasis and metastasis and identifies these as new molecular therapeutic targets that disrupt these processes.

## Results

### NDBT expression is increased in hypoxia heterogeneously

**Fig. 1:**
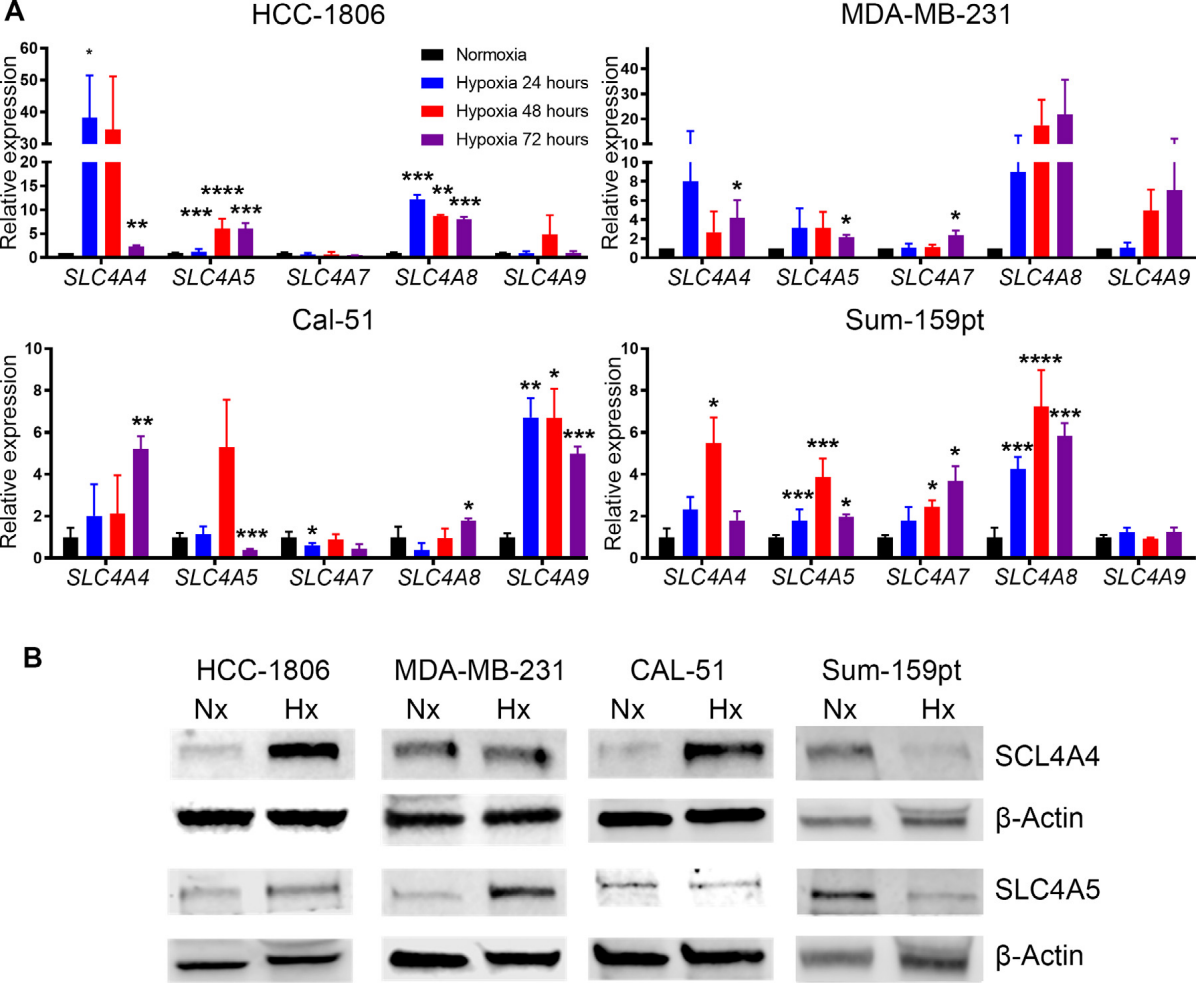
Hypoxia increased the expression of NDBTs (A) QPCR analysis revealed that the expression of NDBT mRNA is increased in hypoxia (O.5% O2) in triple negative breast cancer cell lines. (B) Western blot analysis identified that the expression of SLC4A4 was increased in HCC-1806 and Cal-51 and SLC4A5 in HCC-1806 and MDA-MB-231 in hypoxia. (ANOVA, ***p<0.001, **p<0.01, *p<0.05, significant relative to normoxia, n=3).

The expression of NDBT genes was assessed in normoxia (21% O_2_) and hypoxia (0.5% O_2_) at 24, 48 and 72h by qRT-PCR, in four TNBC cell lines (HCC-1806, MDA-MB-231, CAL-51, SUM159PT)(Fig. 1A). These TNBC cell lines were selected due to being highly responsive to hypoxia (HIF1 stabilization, high CA9 induction), highly migratory *in vitro* and metastatic in vivo. Oxygen levels in breast tumours can vary with a median pO_2_ of 10 mmHg and regions <2.5 mmHg [3]. The hypoxic expression pattern was unique to each cell line; however, the bicarbonate transporters *SLC4A4* (also known as NBCe1), and *SLC4A5* (also known as NBCe2), were the most frequently and most highly upregulated by hypoxia. *SLC4A4* was upregulated significantly with a 5-50-fold increase in 4/4 TNBC cell lines at 48 and/or 72 hours. *SLC4A5* was significantly upregulated with a 4-5-fold increase in 4/4 TNBC cell lines at 48 and/or 72 hours. The protein expression of SLC4A4 and SLC4A5 were investigated in normoxia and hypoxia (0.5% O_2_) at 48 h (Fig. 1B). SLC4A4 expression was significantly increased >4-fold in HCC-1806 and Cal-51 in line with QRT-PCR data but was unchanged in MDA-MB-231 (Fig. S1). SLC4A5 expression was increased >3-fold in HCC-1806 and MDA-MB-231 in line with QRT-RPCR data but was unchanged in CAL-51 (Fig. S1). The protein expression of SLC4A4 and SLC4A5 in Sum-159PT was downregulated in hypoxia and did not correspond with the QRT-PCR data. Analysis of published transcriptomic data using bc-GenExMiner v4.5 (http://bcgenex.centregauducheau.fr/) identified that high expression of SLC4A5 was associated with worse overall survival in basal type breast cancer (Fig. S2).

### NDBT knockdown acidified intracellular pH and disrupted spheroid growth

**Fig. 2:**
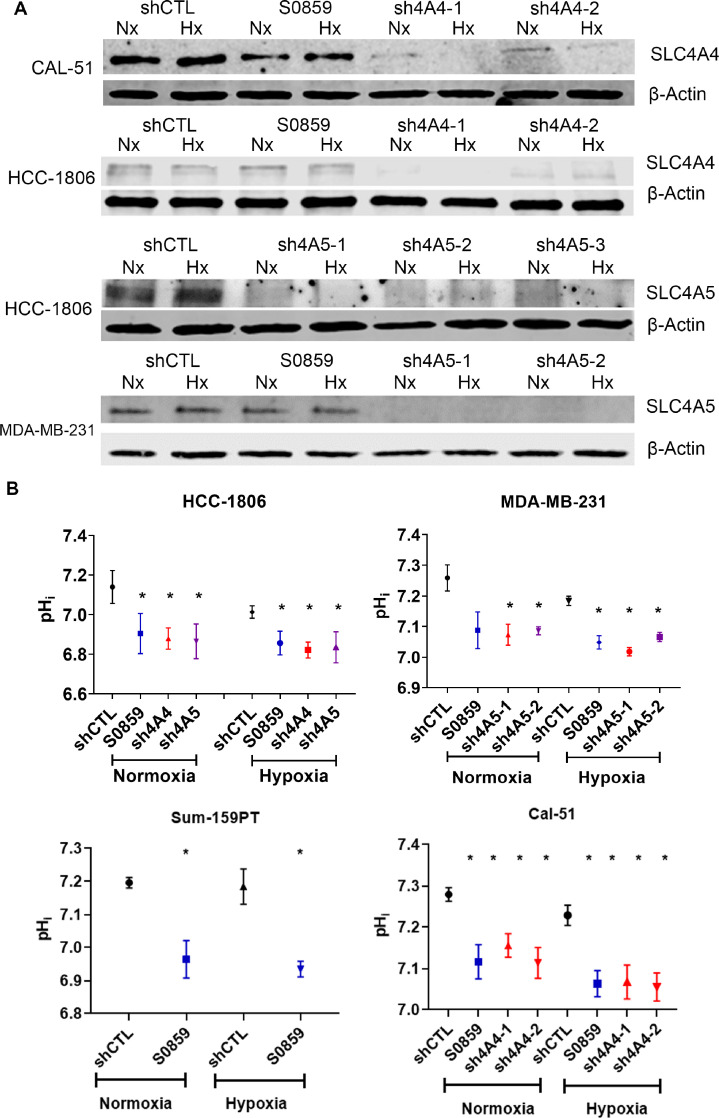
NDBT knockdown or inhibition reduced pHi (A) Knockdown of NDBTs with lentiviral shRNA was validated by western blot in normoxia and hypoxia. (B) NDBT inhibition and knockdown reduced pHi assessed using SNARF^TM^-1 and this effect was further reduced in hypoxia. (shCTL= control shRNA cells, sh4A4/sh4A5= SLC4A4/SLC4A5 targeting shRNA knockdown cells where multiple shRNAs were used to target the same gene this is denoted by -1, -2, -3, S0859=NDBT inhibitor (ANOVA, ***p<0.001, **p<0.01, *p<0.05, significant relative to shCTL, n=3).

SLC4A4 and SLC4A5 were knocked down in TNBC cell lines MDA-MB-231 and HCC1806 using lentiviral shRNA (Fig. 2A). SLC4A4 was knocked-down by >70% at the RNA and protein levels in HCC-1806 and Cal-51 with 3 shRNA sequences (Fig. S3). SLC4A5 was knocked-down in HCC-1806 and MDA-MB-231 using 2-3 shRNA sequences by >80% at the RNA and protein levels (Fig. S3).

We have previously investigated the impact of NDBT knockdown and inhibition on pH regulation in 3D steady state and ammonia prepulse experiments [16]. Here we investigated steady state pH_i_ in NDBT knockdowns/inhibition to identify the pH_i_ impact in the context of 2D culture that we use in our subsequent investigations. Cells were investigated with a pH_i_ sensitive dye in HCC1806, MDA-MB-231, SUM-159PT and Cal-51 (Fig. 2B). Inhibition of NDBT (S0859) significantly reduced pH_i_ in all cell lines (p<0.05, n=3) in hypoxia and in HCC-1806, SUM-159PT and Cal-51 in normoxia (p<0.05, n=3). SLC4A4 knockdown in HCC1806 and Cal-51 significantly reduced pH_i_ in normoxia and hypoxia (p<0.05, n=3). SLC4A5 knockdown significantly reduced pH_i_ in both MDA-MB-231 and HCC1806 in normoxia and hypoxia (p<0.05, n=3)(Fig. 2B). We previously identified that inhibition and knockdown of NDBT reduced spheroid growth and this was validated here and NDBT knockdown/inhibition reduced HCC1806 spheroid growth [16] (Fig. S4).

### NDBT knockdown and inhibition reduces migration and invasion

**Fig. 3:**
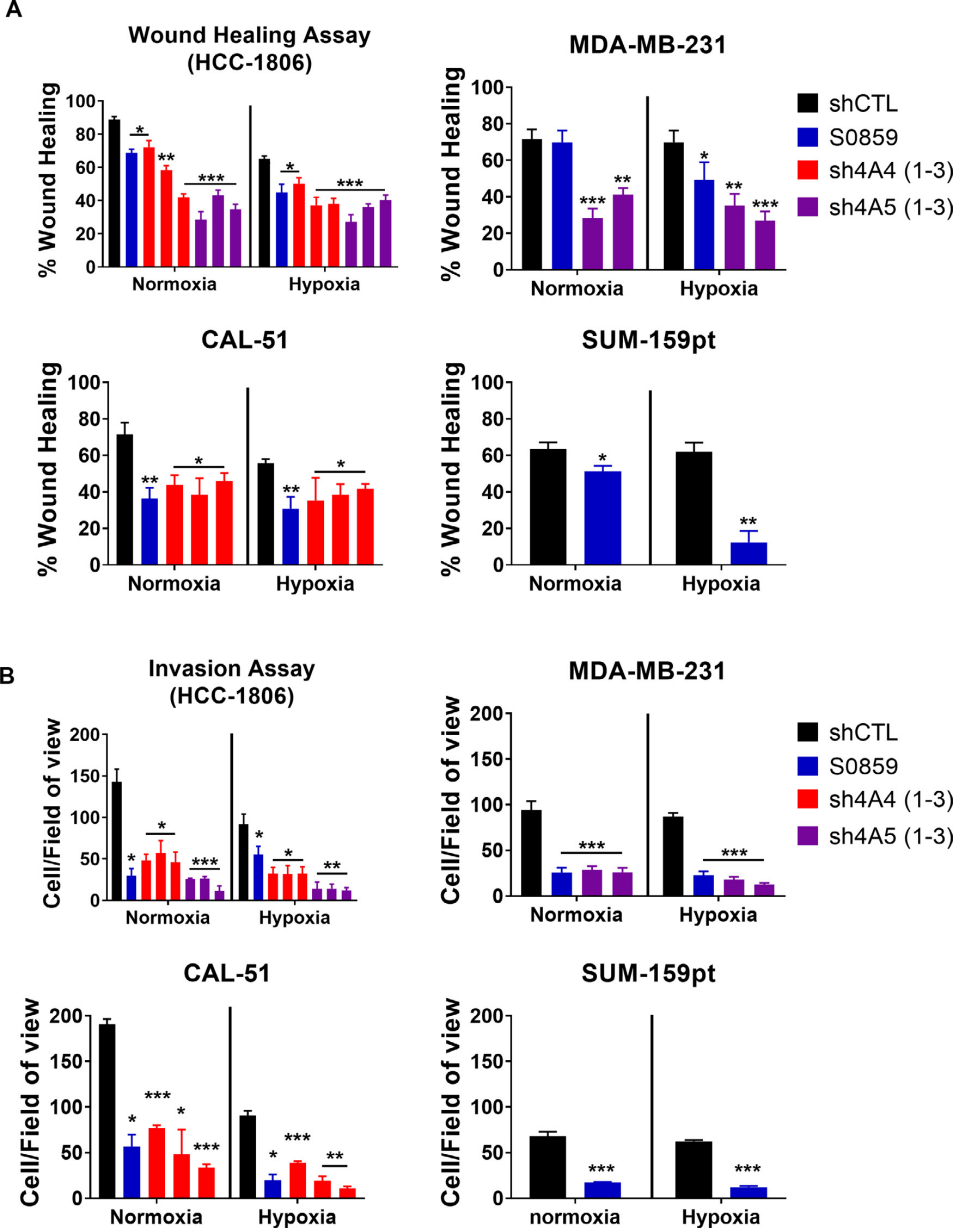
NDBT knockdown or inhibition reduced migration and invasion *in vitro* (A) Wound healing assays were used to identify that NDBT knockdown or inhibition reduces migration in four triple negative breast cancer cell lines in normoxia and hypoxia (1% O_2_). (B) Invasion through a matrigel coated boyden chamber was reduced by NDBT inhibition or knockdown in normoxia and hypoxia in four triple negative cancer cell lines. shCTL=control shRNA cells, sh4A4/sh4A5= SLC4A4/SLC4A5 targeting shRNA knockdown cells where multiple shRNAs were used to target the same gene this is denoted by -1, -2, -3, S0859=NDBT inhibitor (ANOVA/t-test as appropriate, ***p<0.001, **p<0.01, *p<0.05, significant relative to shCTL, n=3).

Migration was assessed using the wound healing assay (Fig. 3A) and invasion was assessed using the modified Boyden chamber assay (Fig. 3B)(Data summarised in SuppT1) in both normoxia and hypoxia. The impact of NDBT inhibition on migration and invasion was investigated in 4 TNBC cell lines (HCC1806, MDA-MB-231, CAL-51 and SUM-159pt) the role of SLC4A4 was investigated in 2 cell lines (HCC1806 and Cal-51) and the role of SLC4A5 was investigated in 2 cell lines (HCC1806 and MDA-MB-231) based on the observed NDBT expression increases in hypoxia.

Inhibition of NDBT (S0859) significantly reduced migration in 3/4 cell lines in normoxia (10-60%)(p<0.05, n=3) and all 4 cell lines in hypoxia (15-50%)(p<0.05)(Fig. 3A). SLC4A4 knockdown significantly reduced migration in normoxia (25-30%)(p<0.05, n=3) and hypoxia (20-27%)(p<0.05, n=3) in both cell lines investigated. SLC4A5 knockdown significantly reduced migration in normoxia (50-60%)(p<0.01, n=3) and hypoxia (42-52%)(p<0.01, n=3) in both cell lines investigated (Fig. 3A)(Data summarised in SuppT1).

Inhibition of NDBT with S0859 significantly reduced invasion in all 4 cell lines in normoxia (60-90%)(p<0.05, n=3) and in hypoxia (50-90%)(p<0.05, n=3)(Fig. 3B). SLC4A4 knockdown significantly reduced invasion in normoxia (50-76%)(p<0.05, n=3) and hypoxia (60%)(p<0.05, n=3) in both cell lines investigated. SLC4A5 knockdown significantly reduced invasion in normoxia (75-86%)(p<0.001, n=3) and hypoxia (70-75%)(p<0.001, n=3) in both cell lines investigated (Fig. 3B)(Data summarised in SuppT1).

### LCK and LYN signalling is perturbed by NDBT knockdown or inhibition

**Fig. 4:**
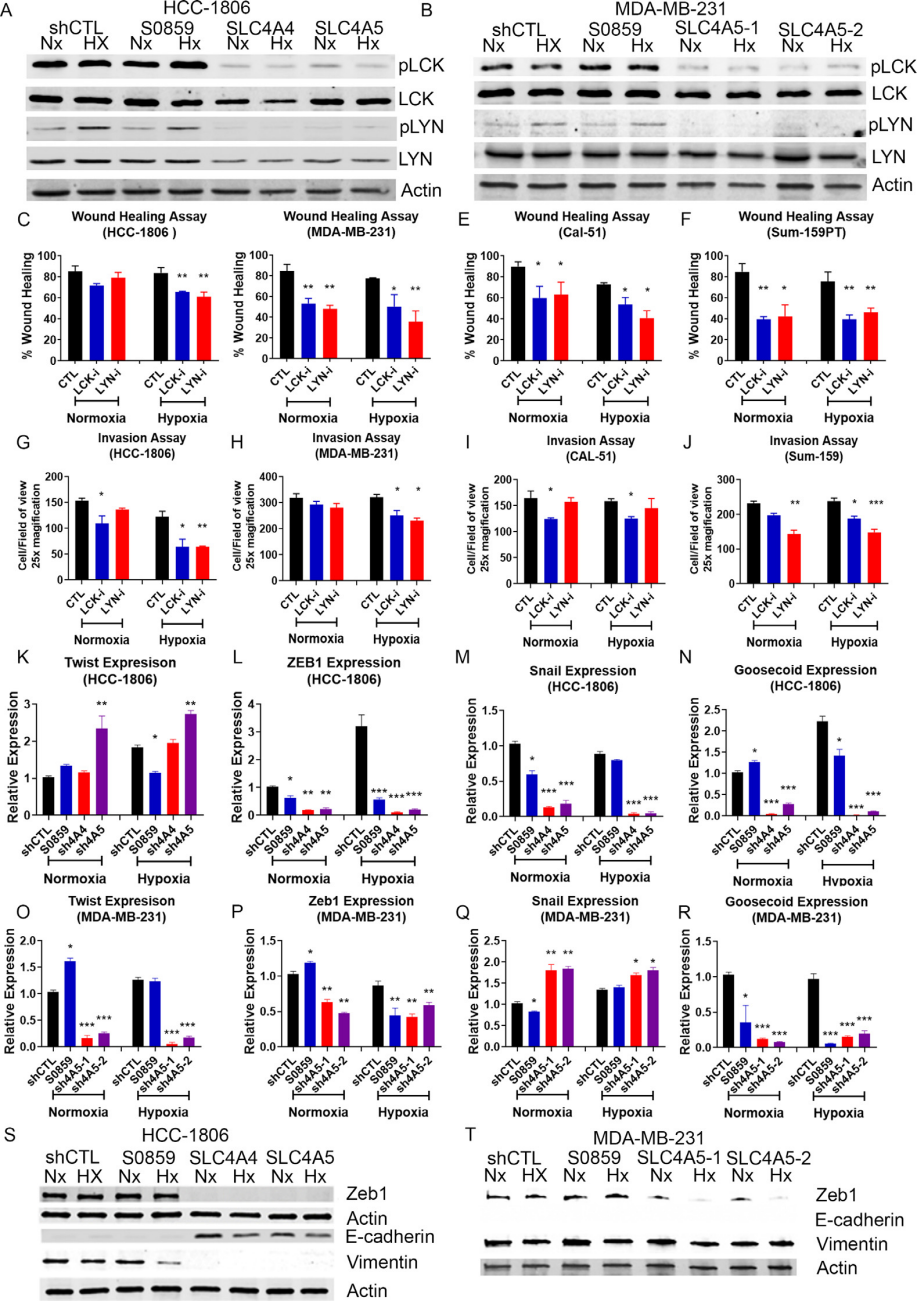
LCK and LYN phosphorylation and EMT are reduced by NDBT knockdown (A-B) Western blot analysis was utilised to validate the phospho-kinase arrays and investigate the expression of LCK and LYN signalling effectors in response to hypoxia and NDBT knockdown. Hypoxia increased phosphorylation of tyrosine kinases LCK and LYN, whereas NDBT knockdown reduces phosphorylation of LCK and LYN. (C-F) Wound healing assays were used to identify that LCK and LYN inhibition reduces migration in four triple negative breast cancer cell lines in normoxia and hypoxia (1% O_2_). (G-J) Invasion through a matrigel coated boyden chambers was reduced by LCK and LYN inhibition in normoxia and hypoxia in four triple negative cancer cell lines. (K-R) QRT-PCR analysis was used to identify that EMT transcription factors (*Twist, ZEB1, Snail* and *Goosecoid*) gene expressions are reduced by NDBT knockdown in normoxia and hypoxia. (S) NDBT knockdown reduces mesenchymal markers Vimentin and ZEB1 expression and increases epithelial marker E-cadherin expression in HCC1806. (T) NDBT knockdown reduces mesenchymal marker ZEB1 expression in MDA-MB-231. shCTL=control shRNA cells, sh4A4/sh4A5= SLC4A4/SLC4A5 targeting shRNA knockdown cells where multiple shRNAs were used to target the same gene this is denoted by -1, -2, -3, S0859=NDBT inhibitor (ANOVA/t-test as appropriate, ***p<0.001, **p<0.01, *p<0.05, significant relative to shCTL, n=3).

To identify the mechanism by which NDBT modulation impacted migration and invasion, we investigated the relationship between NDBTs and phospho-kinase signalling. We performed a preliminary screen to assess if NDBT knockdown or inhibition modulated phospho-signalling using a Human Phospho-Kinase array with the following conditions: shCTL, NDBT inhibition, SLC4A4 knockdown, SLC4A5 knockdown; in neutral (pH 7.4) or acidic (pH 6.4) conditions in hypoxia (0.5%O_2_). Signalling changes regulated at the peak NDBT induction timepoint (48h hypoxia, 0.5% O_2_) were investigated in HCC-1806 cells. Hypoxia increased phosphorylation >20% in 36/43 (83%) proteins (Fig. S5). The largest increases were for LYN (Y397) 6.5-fold and LCK (Y394) 4.3-fold. In addition, the effect of acidosis on was investigated. HCC-1806 was exposed to acidic (pH 6.5) or neutral (pH 7.4) conditions in normoxia for 48h (Fig. S6). In contrast to hypoxia, acidosis reduced phosphorylation and 32/43 (74%) proteins had >20% reduction in phosphorylation including Lyn (Y397) by 90%, and LCK (Y394) by 70% (Fig. S7). Immunoblot validated that hypoxia increased the phosphorylation of LCK by 1.5 fold (p<0.05) and LYN by 4 fold (p<0.01)(Fig. 4A-B). Phosphorylation of LCK and LYN was markedly reduced in response to NDBT knockdown in HCC1806 and MDA-MB-231 in both normoxia and hypoxia (Fig. 4A-B).

### NDBT-regulated phosphorylation of LCK and LYN modulates migration and invasion

LCK (LCK inhibitor IC_50_ 7nM) and LYN (Bafetinib IC_50_ 19nM) inhibitors were used at IC_50_ concentrations to investigate their role in migration and invasion and provide a mechanism by which NDBT knockdown reduces these. In hypoxia, wound healing was reduced 20-50% by LCK inhibition (p<0.05, n=3), and 23-50% by LYN inhibition (p<0.01, n=3)(Fig. 4C-F) in all 4 cell lines, and similar effects were identified in normoxia in 3/4 cell lines. Invasion was reduced by LCK (15-50%)(p<0.05, n=3) and LYN inhibition (30-45%)(p<0.05, n=3) in 3/4 cell lines in hypoxia (Gig4G-J). Less significant effects were all identified for LCK inhibition in 2/4 cell lines (15%)(p<0.05, n=3) and LYN inhibition in 1/4 cell lines (35%)(p<0.01, n=3) in normoxic conditions.

### Epithelial Mesenchymal Transition (EMT) induction is inhibited by NDBT knockdown

Hypoxia and acidosis can alter EMT gene expression [27,28]. Given the impact of NDBT knockdown/inhibition on migration, invasion and phospho-signalling we investigated whether NDBT knockdown perturbed EMT. The expression of key EMT genes was investigated by qRT-PCR (Fig. 4K-R). NDBT knockdown or inhibition altered EMT gene expression in HCC1806 (Fig. 4K-N) and MDA-MB-231 (Fig. 4O-R). Snail, *Zeb1*, and *goosecoid* were significantly downregulated by NDBT knockdown in both cell lines in normoxia and hypoxia (Fig. 4L-N, P-R). Twist expression was significantly downregulated by SLC4A5 knockdown in MDA-MB-231 but was upregulated by SL4A5 knockdown in HCC1806. The impact of NDBT inhibition on EMT gene expression largely followed the pattern identified with NDBT knockdown with the exception of *Twist* expression in HCC1806 and MDA-MB-231, *Snail* expression in MDA-MB-231 in normoxia and Goosecoid expression in HCC1806 in normoxia. The difference in expression regulation between NDBTs and knockdown and inhibition highlights heterogeneity of response to pH_i_ modulation in EMT gene regulation. Knockdown of NDBTs decreased the expression of EMT regulating protein ZEB1 (Fig. 4S-T, Fig. S8) in normoxia and hypoxia in HCC1806 and MDA-MB-231 cell lines, but this was unaltered by NDBT inhibition. Knockdown of NDBTs increased the expression of epithelial marker E-cadherin and reduced the expression of mesenchymal marker Vimentin in HCC1806 in normoxia and hypoxia (Fig. 4S, Fig. S8).

### Metabolic homeostasis is altered by NDBT knockdown

**Fig. 5:**
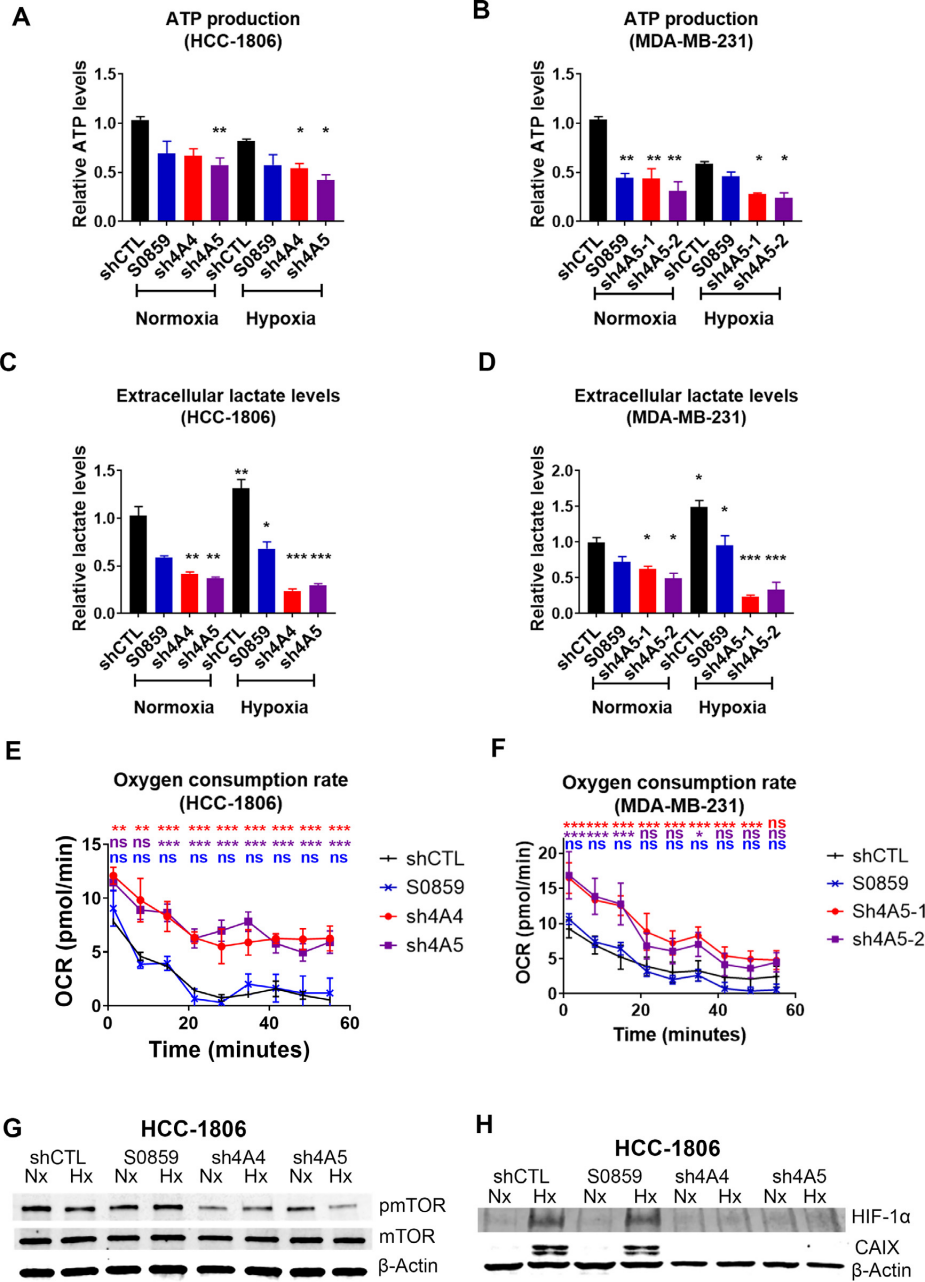
NDBT knockdown and inhibition reduced ATP and lactate levels but increases oxygen consumption in hypoxia (A-B) ATP levels are reduced by NDBT knockdown and inhibition in normoxia and hypoxia (1% O_2_). (C-D) NDBT knockdown or inhibition reduced extracellular lactate levels in normoxia and hypoxia (1% O_2_). (E-F) NDBT knockdown increased oxygen consumption rates in DMOG treated HCC1806 and MDA-MB-231 as assessed by Seahorse real-time cell metabolic analysis. (G) Activation of mTOR is reduced by NDBT knockdown in normoxia and hypoxia (HCC1806). (H) Stablisation of HIF-1α, and the expression of the HIF-1α transcriptional target CA9, are reduced in hypoxia by NDBT knockdown (HCC1806). shCTL=control shRNA cells, sh4A4/sh4A5= SLC4A4/SLC4A5 targeting shRNA knockdown cells, S0859=NDBT inhibitor (ANOVA, ***p<0.001, **p<0.01, *p<0.05, ns = not significant, n=3).

Increased ATP production is required during metastasis [29]. Kinase signalling controls many aspects of metabolism and was perturbed by NDBT knockdown; therefore, we investigated metabolic regulation by NDBT. In HCC1806 ATP production was reduced by SLC4A5 knockdown (40%, p<0.01, n=3)(Fig. 5A) in normoxia and by SLC4A4 (40%, p<0.05, n=3) and SLC4A5 (50%, p<0.05, n=3)(Fig. 5A) in hypoxia. In MDA-MB-231 ATP production was reduced by NDBT inhibition (25%, p<0.05, n=3), SLC4A4 knockdown (40%, p<0.05, n=3), an SLC4A5 knockdown (50%, p<0.01, n=3)(Fig. 5B) in normoxia and by SLC4A4 (40%, p<0.05, n=3) and SLC4A5 (50%, p<0.05, n=3)(Fig. 5B) in hypoxia.

Glycolysis is a major source of ATP production in hypoxia therefore we investigated the impact of NDBT inhibition and knockdown on lactate production. Lactate levels were increased in response to hypoxia (>35%, p<0.05, n=3) in both HCC1806 and MDA-MB-231(Fig. 5C-D). In HCC1806 extracellular lactate levels were reduced in normoxia by SLC4A4 (45%, p<0.01, n=3), and SLC4A5 knockdown (50%, p<0.01, n=3) and to a greater extent in hypoxia by SLC4A4 knockdown (75%, p<0.001, n=3), and SLC4A5 knockdown (70%, p<0.001, n=3)(Fig. 5C). In MDA-MB-231 extracellular lactate levels were reduced in normoxia by SLC4A4 (35%, p<0.05, n=3), and SLC4A5 knockdown (45%, p<0.05, n=3) and to a greater extent in hypoxia by SLC4A4 knockdown (80%, p<0.001, n=3), and SLC4A5 knockdown (75%, p<0.001, n=3)(Fig. 5D).

As mitochondrial respiration is also key in ATP production the oxygen consumption rate was assessed. The oxygen consumption rate was higher in NDBT knockdown cells in both HCC1806 and MDA-MB-231 (p<0.01, n=3)(Fig. 5E-F). This effect was also seen in DMOG-treated cells. The extracellular acidification rate was unchanged by NDBT knockdown or inhibition (Fig. S9).

The phospho-kinase array (Fig. S7) identified that the major regulator of metabolism mTOR activating phosphorylation (S2448) was reduced by NDBT knockdown. As NDBT knockdown modulated metabolism we validated this result by immunoblot which identified that phosphorylation of mTOR was reduced by SLC4A4 (70%) and SLC4A5 (80%) knockdown in hypoxia (Fig. 5G)(HCC1806). mTOR regulates HIF stabilization in hypoxia [24] and we identified that NDBT knockdown ablated HIF-1α stabilisation and expression of HIF-1α target CA9 in hypoxia (Fig. 5H)(HCC1806). NDBT inhibition (S0859) did not substantially impact mTOR phosphorylation, HIF stabilisation or CA9 expression. The loss of HIF-1α stabilisation in hypoxia in response to NDBT knockdown is in line with the increased hypoxic oxygen consumption rates seen in DMOG treated cells (Fig. 5E-F).

### NDBT knockdown reduces metastatic colonisation

**Fig. 6:**
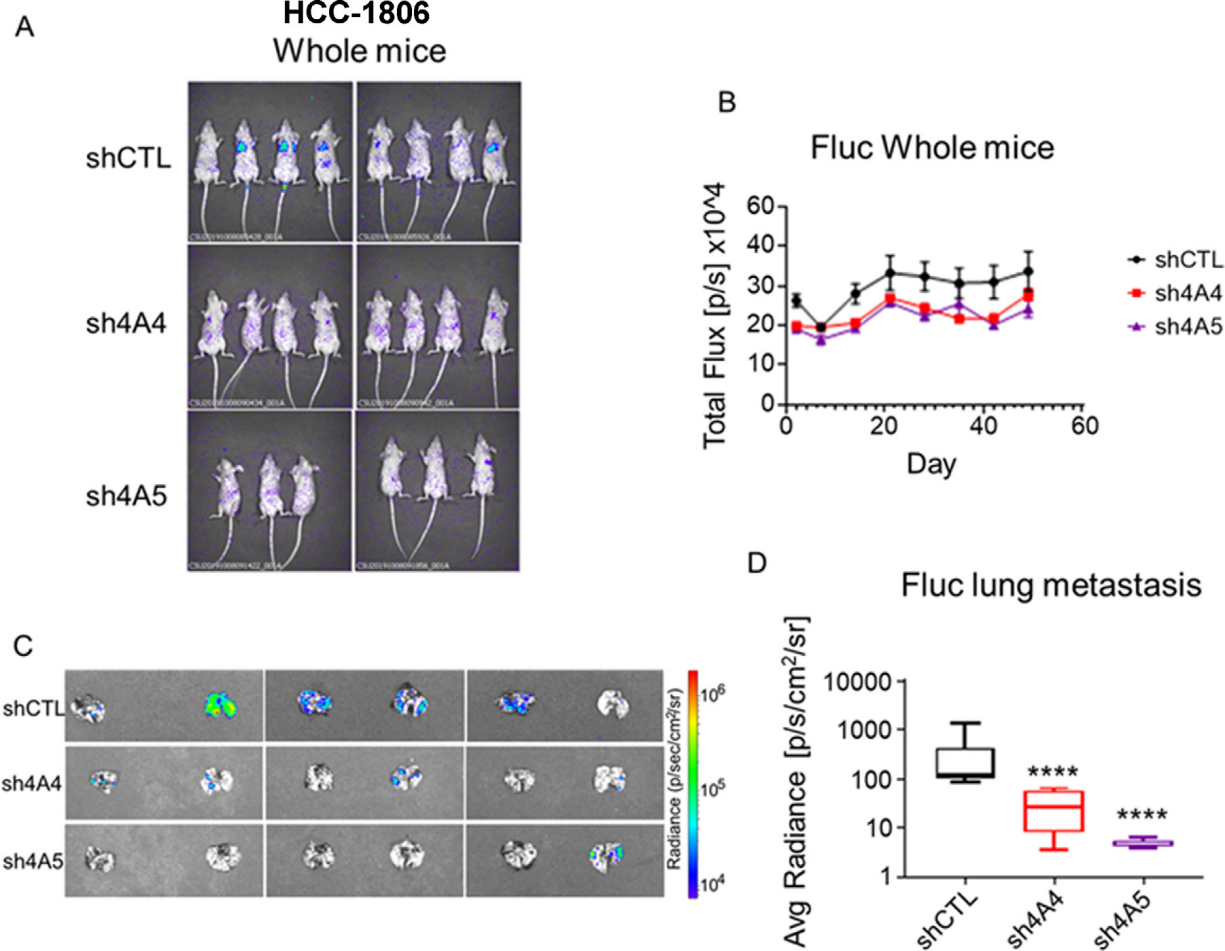
NDBT knockdown reduced metastatic colonisation *in vivo* (A) HCC-1806 cells were injected into SCID mice via tail vein injection. Luciferase expression was reduced by NDBT knockdown prior to termination. (B) Total metastatic growth was reduced by NDBT knockdown. (C-D) Lung colonisation was reduced by NDBT knockdown. shCTL=control shRNA cells, sh4A4/sh4A5= SLC4A4/SLC4A5 targeting shRNA knockdown cells, S0859=NDBT inhibitor (****p<0.0001, significant relative to shCTL, n=6-8).

The role of NDBT in the regulation of metastatic potential was assessed *in vivo*. We previously identified a significant reduction in tumour xenograft growth rate in response to NDBT knockdown [16]. In this study the tail vain injection model was selected over other metastatic models to prevent the impact of NDBT knockdown on tumour growth confounding results where reduced metastasis could be due primarily to reduced primary tumour volume. Direct injection into the tail veil allowed investigation of metastatic dissemination and colonisation only. Metastasis was reduced by NDBT knockdown (Fig. 6A-D). Lung metastasis as detected via luciferase imaging of lungs and was reduced by 94% (p<0.001, n=8) by SLC4A4 knockdown and 98% (p<0.001, n=6) by SLC4A5 knockdown (Fig. 6C-D).

## Discussion

The hypoxic tumour microenvironment places metabolic stress on cells requiring adaption to the increased acidity commonly associated with hypoxic tumour regions[16]. HIF stabilisation upregulates many pH regulatory proteins [30]. Hypoxic tumours may therefore be sensitive to a therapeutic strategy targeting these proteins and interrupting pH regulation. We previously identified the role of hypoxia-regulated NDBT in regulation pH_i_ and that NDBTs knockdown/inhibition reduced spheroid growth, increased apoptosis at the core of spheroids and reduced xenograft growth rates [16].

Here we investigated the role of hypoxia regulated NDBT on metastatic phenotypes, and the mechanism driving NDBT regulation of these processes in TNBC. 0.5% O_2_ hypoxia was used experimentally as a physiologically-relevant partial pressure for breast tumours which has been shown to be between 0.1% and 1% O_2_ [31]. We identified a heterogeneous pattern to NDBT upregulation in TNBC cell lines as was shown previously in other tumour types (Fig. 1A) [16]. No correlation between phenotype and particular NDBT upregulation was seen, where SLC4A4 and SLC4A5 knockdown produced similar phenotypic effects in experiments.

We generated shRNA knockdowns of NDBT (Fig. 2A) and identified that NDBT knockdown and inhibition reduced steady state pH_i_ in all 4 cell lines investigated in line with our previous observations (Fig. 2B) [16]. A key result from this study is the reduced migration and invasion observed by NDBT knockdown and inhibition that was seen in all 4 cell lines investigated in normoxia and hypoxia (Fig. 3). This is in concordance with other pH regulatory proteins which modulate migration and invasion and metastasis; including CA9, MCT4 and NHE1 [32,33].

We investigated possible mechanisms by which pH_i_ regulation by NDBT could modulate the migratory and invasive properties of TNBC. We showed regulation of tyrosine-kinase phosphorylation by NDBT knockdown/inhibition in hypoxia (Fig. 4A-B, Fig. S7). This is likely due to increased protonation of histidine residues on proteins as pH_i_ acidifies, perturbing kinase function [34]. The regulation of metastatic phenotypes by protein tyrosine-kinase signalling is well-established [35]. We showed significant changes in phosphorylation states of LYN and LCK in hypoxia and acidosis (Fig. 4A-B, Fig. S6). LCK and LYN are kinases of the SRC family [36]. In TNBC they are activated more frequently than metastatic regulators SRC and FAK [37]. Both LCK and LYN regulate migration and inhibition and LYN reduces breast cancer metastasis [38]. There is little data on the roles of LCK and LYN in hypoxia. Multiple LCK and LYN inhibitors are available, however the only clinically utilised inhibitor is bafetinib [38], which more effectively inhibits BCR-ABL which isn't expressed in these cells [38]. Here both LCK and LYN inhibitors had a significant effect in invasion and migration in hypoxia as well as normoxia (Fig. 4C-J).

EMT plays an important role in breast cancer metastasis, altering morphology and activating migration and invasion [39]. LCK regulates EMT [36] and extracellular acidosis promotes EMT by inducing the expression of transcriptions factors Twist and Snail [40]. We investigated the impact of NDBT knockdown and identified that this inhibits EMT reducing the expression of key EMT genes including *Twist, ZEB1, Snail* and *Goosecoid* (Fig. 4K-R). The expression of EMT regulator ZEB1 protein was reduced in response to NDBT knockdown (Fig. 4S-T)(Fig. S8).The reduction of expression of these EMT transcription factors provides a mechanism by which NDBT knockdown results in reduced migratory and invasive properties. Furthermore the mesenchymal marker Vimentin was reduced and the epithelial marker E-cadherin was increased in response to NDBT knockdown in HCC1806 (Fig. 4S)(Fig. S8).

During EMT and metastasis cells become more glycolytic and are reliant on ATP to support the energy requirements of morphological changes that drive migration, invasion and metastasis [41]. NDBT knockdown reduced ATP (Fig. 5A-B) and lactate levels (Fig. 5C-D) indicating glycolysis is affected by intracellular acidosis. Furthermore, the oxygen consumption rate was higher in NDBT knockdowns treated with DMOG (Fig. 5E-F)(a hypoxia mimetic, that stabilizes HIF by inhibiting PHD proteins). This indicates that a metabolic shift occurs due to NDBT targeting. To identify a mechanism by which NDBT targeting could impact metabolism we investigated key metabolic regulators mTOR and HIF-1α [42,43]. We showed that NDBT knockdown or inhibition reduced mTOR activation (Fig. 5G). mTOR is a known regulator of HIF stabilisation via modulation of protein synthesis and HIF-1α was also reduced by NDBT knockdown in hypoxia (Fig. 5H). Together the reduction of mTOR phosphorylation and HIF-1α stabilisation by NDBT targeting will impact the metabolic and other phenotypic processes that drive metastasis in TNBC [42].

Finally, to validate the *in vitro* analysis we investigated the impact of Knockdown of SLC4A4 and SLC4A5 on metastasis *in vivo*. We used the tail vain injection model and not other metastatic models to prevent the impact of NDBT knockdown on tumour growth, that we have previously shown *in vivo* [16], confounding results where reduced metastasis could be due primarily to reduced primary tumour growth. SLC4A4 and SLC4A5 knockdown significantly reduced metastatic colonisation of the lung (Fig. 6C-D). The lungs are a common metastatic site of TNBC, and metastasis is the cause of 90% of breast cancer deaths. Reducing metastasis could significantly increase life expectancy. Our data identify that targeting NDBTs could be used as a therapeutic approach to reducing TNBC metastasis. The reseeding of metastatic sites and the role of circulating tumour cells in this process after tumour excision indicates that this approach may be of value.

Given the possible roles of NDBT in normal physiology it is important to consider toxicity. We previously reviewed possible toxicities associated with targeting NDBT [16], in summary NDBT knockout mice and human patients with NDBT inactivating mutations exhibit a range of health problems which largely have a developmental basis [44], [45], [46], [47] and therefore targeting NDBTs may still be viable. Alternatively, the production of a hypoxia activated prodrug to target NDBT in tumours may be a realistic therapeutic option to reduce toxicity. A further consideration is that the immune response is also sensitive to pH, where a low pH is pro-inflammatory. Combining NDBT inhibition with immune checkpoint inhibitors such as anti-PD1-PDL1 therapy could potentially produce a synergistic effect for hypoxic cells. The NDBT inhibitor S0859 produced weaker results than knockdown in some experiments and this may be due to a lack of specificity and off target effects [48]. Currently there is no clinically-relevant inhibitor of NDBT, and this research highlights the importance of developing an approach to targeting NDBTs clinically. Targeting NDBT could provide an approach to reducing cancer metastasis and increasing patient survival.

## Materials and Methods

### Cell culture

Cell lines maintained in DMEM supplemented with 10% FBS were available from ATCC (HCC-1806, MDA-MB-231) and Creative Bioarray (CAL-51, SUM-159pt). Cell lines were authenticated by STR analyses and mycoplasma tested (MycoAlert^TM^, Lonza) at regular intervals. Cells were maintained in a humidified incubator at 5% CO_2_ and 37°C and hypoxic exposure was at either 1% or 0.5% (BakerRuskinn, InvivO_2_). Normoxia was defined as 21% O_2_ 5% CO_2_. Acidosis media was prepared as previously [16]. Spheroid aggregation was initiated with 10,000 cells in ultra-low–adherent round-bottom 96-well plates followed by centrifugation at 2000 × g. Clonogenic assays were performed as previously [16]. To knockdown *SLC4A4* or *SLC4A5* in HCC-1806, Cal-51, and MDA-MB-231, shRNA lentivirus was purchased from Sigma-Aldrich (Supplementary Table 2). Cells were grown under Puromycin (Gibco) selection (HCC-1806 and MDA-MB-231 2 μg/mL; Cal-51, 10 μg/mL) to select shRNA expressing cells. Experiments were optimised and normalised by cell number at the end of experiments.

### Chemicals

S0859 (#SML0638, Merk) was reconstituted in DMSO (100µM) in line with others and our previous studies and [16]. Bafetinib (S1369, Seleckchem) was used at 19nM (IC_50_). LCK inhibitor (#79335, Cayman) was used at 7nM (IC_50_).

### Intracellular pH measurements

pH was assessed using 1x staining solution SNARF™-1 (Invitrogen, c1270) according to manufacturer's instructions. Cells were imaged using confocal microscopy, excited at 488nm and emission at 580nm and 640nm. A nigericin standard curve was used as described previously [49].

### Immunoblotting

Cell lysates separated by 10% SDS-PAGE (BioRad) were transferred to nitrocellulose membrane. Antibodies are denoted in Supplementary Table 2. Co-IP was performed using 2mg of cell lysate using Sera-Mag beads (BioRad).

### Quantitative PCR

RNA was extracted using Trizol (Sigma-Aldrich) and cDNA generated using Superscript (ThermoFisher) according to manufacturer's instructions. Quantitative PCR was completed as described previously [13]. Data were normalized to the control gene ACTB. Shapiro–Wilk test were used to determine normality. Data variance shown as standard error of mean. Primer sequences are available in Supplementary Table 3.

### Wound Healing

Cells were seeded to give 100% confluence (20,000 cells/well) in a 24 well plate (Greiner, 662102) at 24h. Cultures were scratched to produce a single wound per well. Wounds were washed with culture medium to remove debris. Images were taken at 0 h and 24 h post scratch and wound healing calculated as the percentage change using Fiji.

### Invasion

Boyden chambers (8 µM pore, Greiner, 662638) were coated with 50ul 1mg/ml Matrigel (Corning, 356255). 100,000 cells were seeded onto the upper chamber in 1% FBS DMEM. 10% FBS DMEM was placed into the lower chamber. Cells were incubated for 24h, fixed with methanol and stained with 0.5% crystal violet (FisherScientific, AC405830250). Cells were manually counted.

### Phospho-signalling array

Phospho-kinase levels were analysed using the Human Phospho-Kinase Array (R&D Systems, ARY003C) according to manufacturer's instructions. Membranes were imaged using a Licor (Odessey) and pixel density analysed using image studio.

### ATP, and lactate assay

The ATP assay (Abcam, ab83355) was performed according to manufacturer's instructions. Luminescence was quantified on FLUOstar Omega. The Lactate assay (Promega, J5021) was performed according to manufacturer's instructions.

### Seahorse assay

Oxygen consumption and extracellular acidification rate assays were performed to manufacturer's instructions in the presence of CO_2_ and HCO_3_ (Agilent, 103693-100). Cells were seeded at 10,000 cells per well and normalised to cell number at the end of the experiment. Full buffer composition can be found in supplementary methods.

### Xenograft

Procedures were carried out under Home Office licence (P435A9CF8) under the supervision of licensed technicians. Shapiro–Wilk test were used to determine normality. Full details can be found in supplementary methods.

### Analysis of the prognostic associations of NDBT in breast cancer

The prognostic associations of NDBT gene expression were investigated using bc-GeneExMiner online tool (http://bcgenex.ico.unicancer.fr/) in n=1980 samples which were investigated in the Molecular Taxonomy of Breast Cancer International Consortium (METABRIC) data set.

## CRediT authorship contribution statement

**Christopher Paul Carroll:** Visualization, Investigation, Data curation, Formal analysis, Writing – original draft. **Hannah Bolland:** Validation. **Eric Vancauwenberghe:** Validation. **Pamela Collier:** Methodology, Investigation, Data curation. **Alison A. Ritchie:** Methodology, Investigation. **Philip A. Clarke:** Methodology, Software, Resources. **Anna M. Grabowska:** Supervision, Conceptualization, Resources. **Adrian L Harris:** Supervision. **Alan McIntyre:** Conceptualization, Methodology, Software, Resources, Writing – review & editing, Supervision, Project administration, Funding acquisition.

## Appendix Supplementary materials

Image, application 1
