# Supplementary material for: Targeting hypoxia regulated sodium driven bicarbonate transporters reduces triple negative breast cancer metastasis
Source: Neoplasia. 2022 Feb 9;25:41–52. doi: 10.1016/j.neo.2022.01.003 (PMC8844412; doi:10.1016/j.neo.2022.01.003)
Supplement: Supplementary file 1 [file mmc1.docx]

Supplementary Data


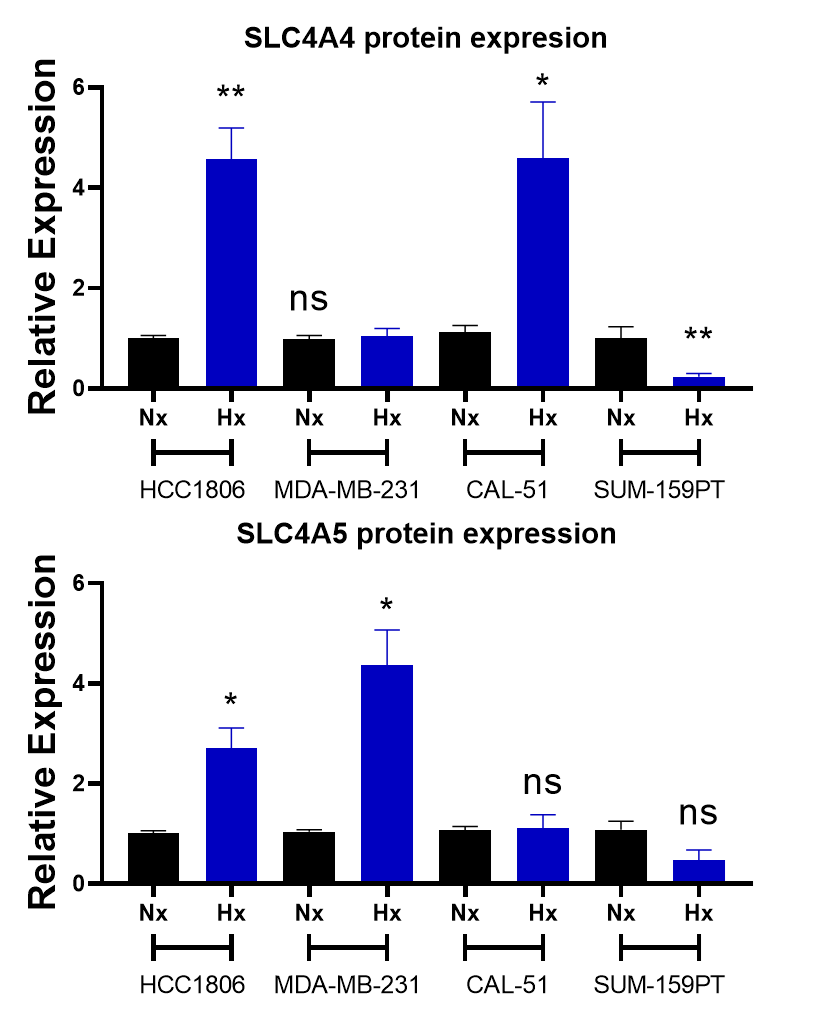


**Figure S1. Protein expression of NDBTS is increased in hypoxia in a heterogenous manner.**


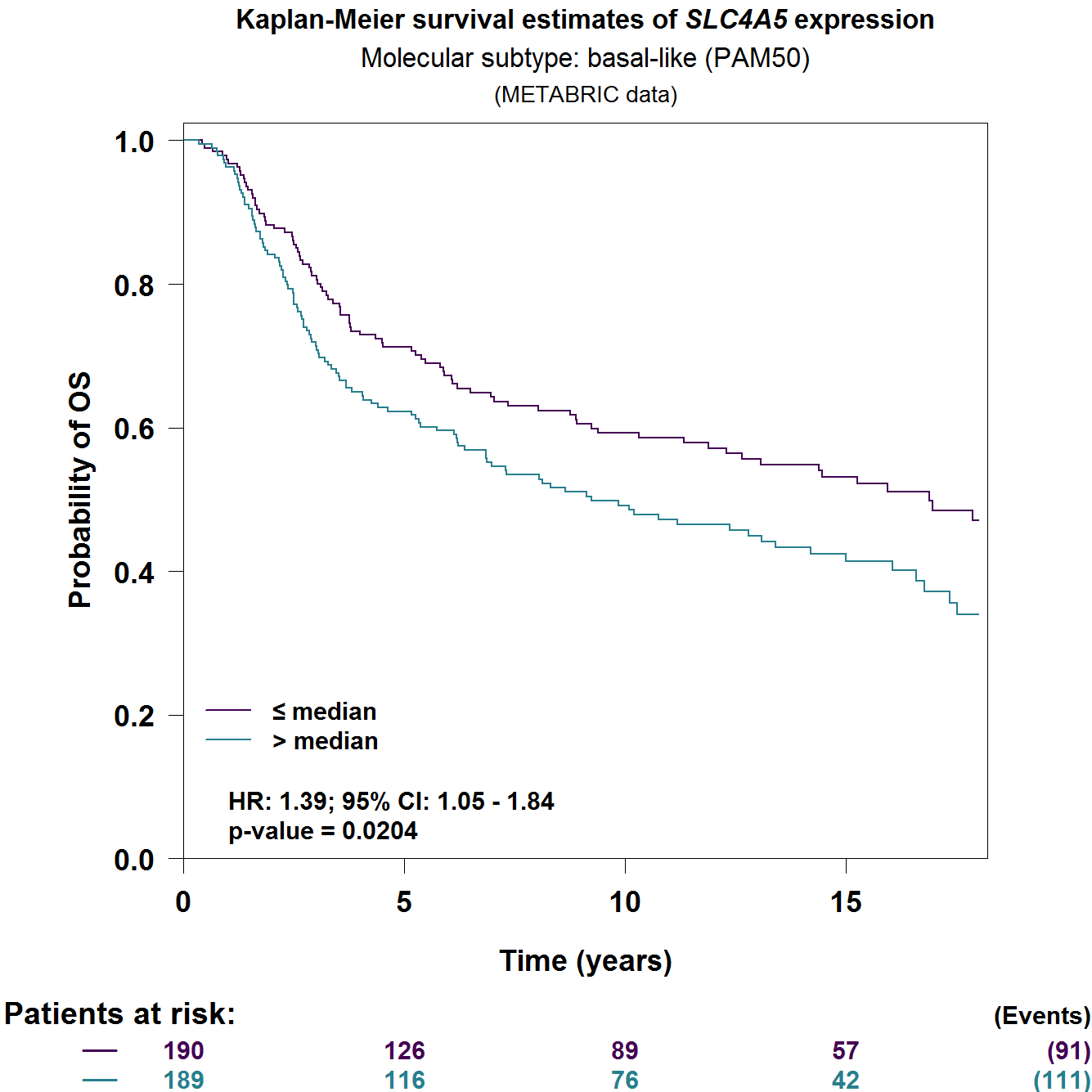


**Figure S2. High expression of SLC4A5 in basal like breast cancer is associated with worse patient survival**Kaplan Meier curve analysis of the correlation between SLC4A5 expression and overall survival in basal like breast cancer is associated with worse patient survival using Breast Cancer Gene-Expression Miner v4.5 (http://bcgenex.centregauducheau.fr/).


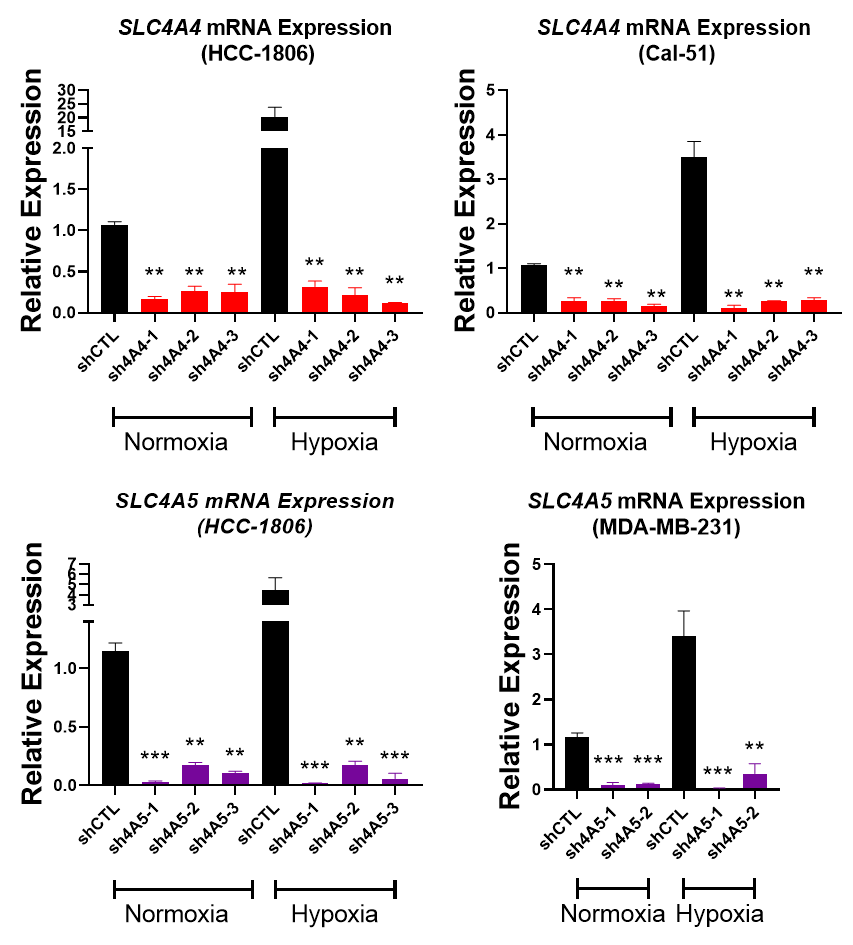


**Figure S3. NDBT mRNA expression is reduced following transduction of shRNA**


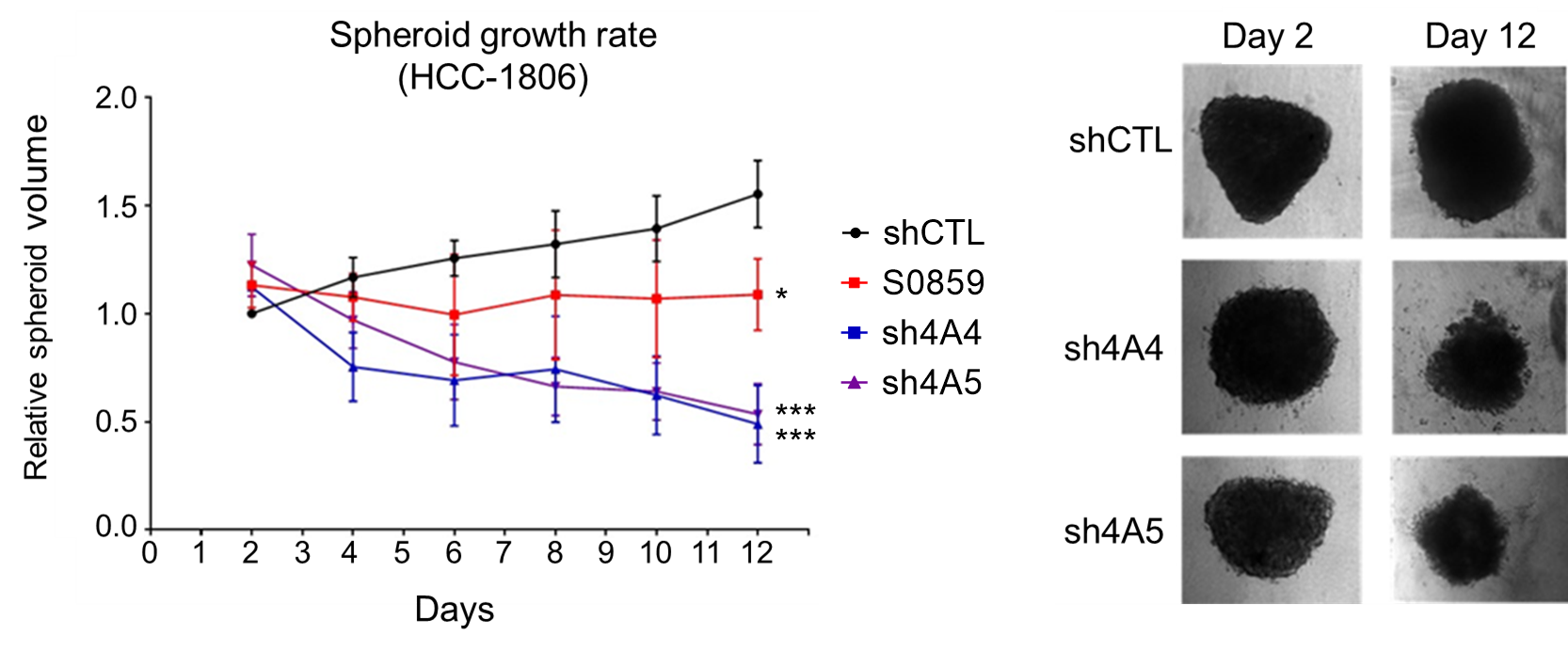


**Figure S4. NDBT knockdown or inhibition reduces spheroid growth rate in HCC-1806**Spheroid growth was measured in HCC-1806 cells over 12 days. Inhibition of NDBTs resulted in no spheroid growth. Knockdown of SLC4A4 and SLC4A5 reduced spheroid size and induced necrosis. shCTL=control shRNA cells, sh4A4/sh4A5= SLC4A4/SLC4A5 targeting shRNA knockdown cells, S0859=NDBT inhibitor. (ANOVA, ***p<0.001, *p<0.05, significant relative to shCTL, n=3).

**Figure S5. Hypoxia increases protein tyrosine kinase phosphorylation**Hypoxia (0.5%, 48h) induced an increase in protein tyrosine kinase phosphorylation in HCC-1806. Increased phosphorylation was observed in 40 out of 43 proteins on the Human Phospho-Kinase Array (R&D Systems, ARY003C). The results show the intensity values.

**Figure S6. Acidosis reduces protein tyrosine kinase phosphorylation**Acidosis (pH 6.5, 48h) reduced phosphorylation of protein tyrosine kinases in HCC-1806 as assessed by the Human Phospho-Kinase Array (R&D Systems, ARY003C). The results show the intensity values.

**Figure S7. Protein tyrosine kinase phosphorylation is reduced by NDBT knockdown in hypoxia and acidosis**NDBT knockdown or inhibition reduces the phosphorylation of protein tyrosine kinases in hypoxia and neutral pH assessed using the Human Phospho-Kinase Array (R&D Systems, ARY003C). Hypoxia and acidosis reduces the induction of phosphorylation of protein tyrosine kinases when compared to hypoxia and neutral conditions. Hypoxia and acidic pH combined with NDBT knockdown or inhibition results in a further reduction of phosphorylation. shCTL=control shRNA cells, sh4A4/sh4A5= SLC4A4/SLC4A5 targeting shRNA knockdown cells, S0859=NDBT inhibitor. The results show the intensity values.


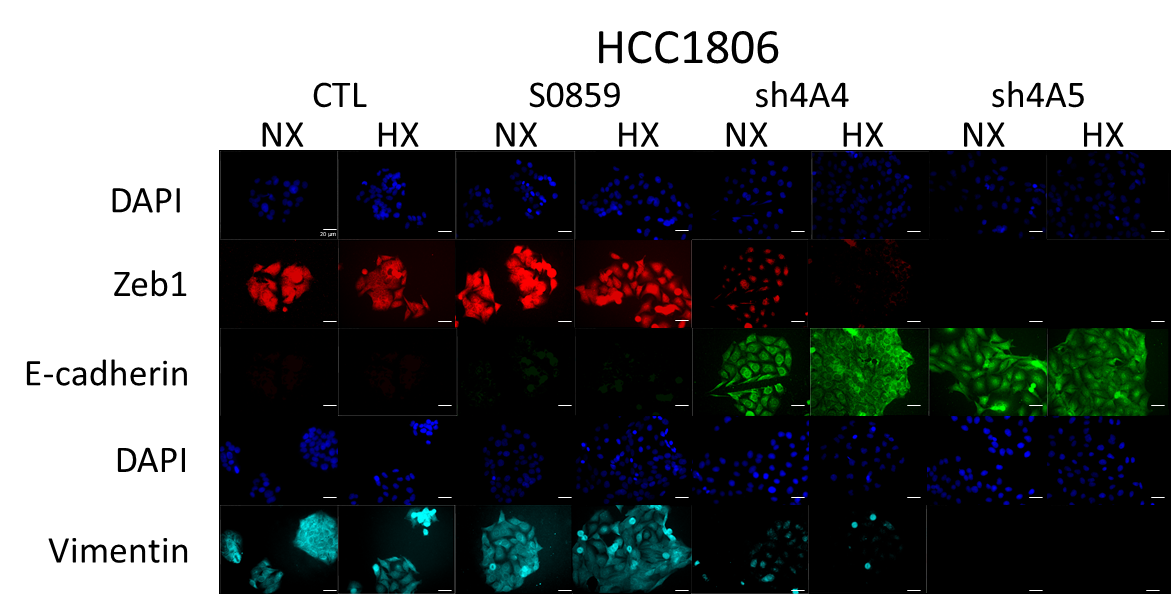


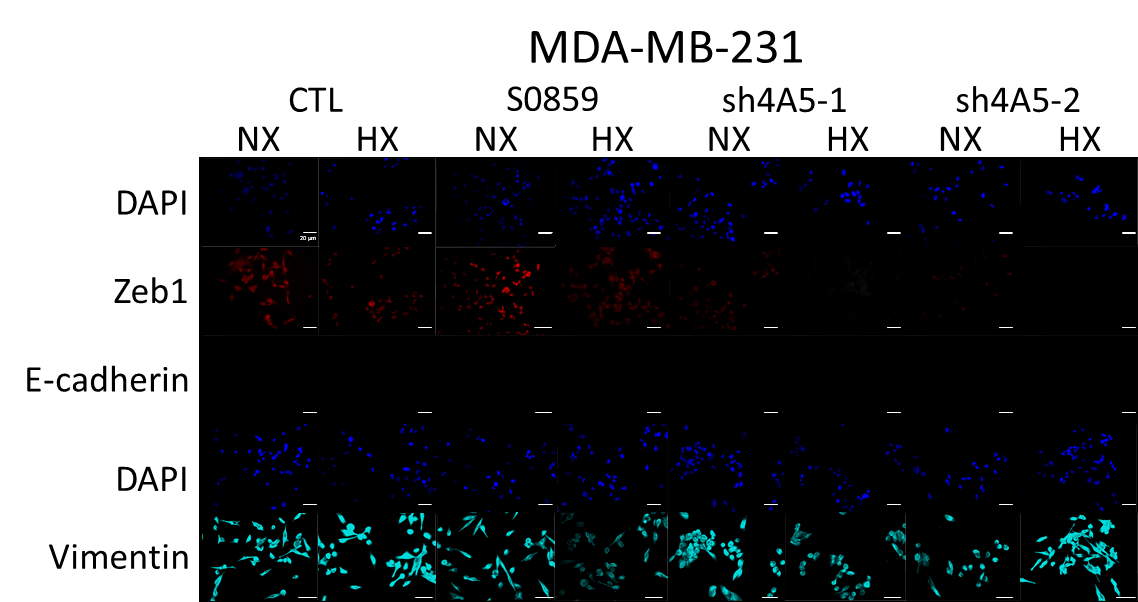


**Figure S8. Knockdown of NDBTs reduced the expression of EMT transcription factors**

Knockdown of NDBTs decreased the expression of EMT regulating protein ZEB1 in normoxia and hypoxia but this was unaltered by NDBT inhibition. Knockdown of NDBTs increased the expression of epithelial marker E-cadherin and reduced the expression of mesenchymal marker Vimentin in normoxia and hypoxia.CTL=control shRNA cells, sh4A4/sh4A5= SLC4A4/SLC4A5 targeting shRNA knockdown cells, S0859=NDBT inhibitor. The results are representative immunofluorescence images (n=3). Scale bar=20uM


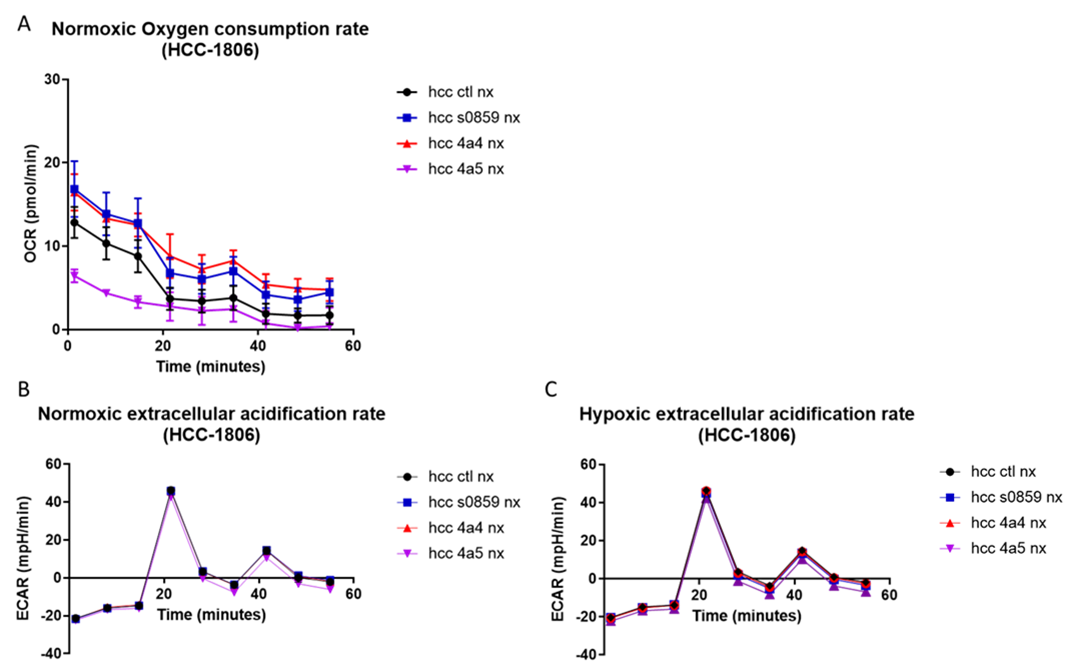


**Figure S9. Seahorse real-time cell metabolic analysis revealed that NDBT knockdown does not alter oxygen consumption in normoxia**

(A) Oxygen consumption is not altered by NDBT knockdown or inhibition in normoxia. (B, C) Extracellular acidification rate was not altered by NDBT knockdown or inhibition in either normoxia or hypoxia. shCTL=control shRNA cells, sh4A4/sh4A5= SLC4A4/SLC4A5 targeting shRNA knockdown cells, S0859=NDBT inhibitor.

**Table S1. Summary of Migration and Invasion data for NDBT inhibition and knockdown in HCC1806, MDA-MB-231, CAL-51 and SUM-195pt.**

**Table S2 lentivirus sequences**

| **Product ID** | **Name** | **Sequence** |
| --- | --- | --- |
| SHC016V | MISSION® pLKO.1-puro Non-Target shRNA Control Transduction Particles Targets no known genes from any species | CCGGGCGCGATAGCGCTAA TAATTTCTCGAGAAATTATTA GCGCTATCGCGCTTTTT |
| NM_003759.1-3183s1c1 | TRCN0000043141 | CCGGCCTTTCCTAAGCGATA GCAAACTCGAGTTTGCTATC GCTTAGGAAAGGTTTTTG |
| NM_003759.1-2376s1c1 | TRCN0000043138 | CCGGCCGGCTTTGTTGGTCA CTATACTCGAGTATAGTGAC CAACAAAGCCGGTTTTTG |
| NM_003759.1-1955s1c1 | TRCN0000043140 | CCGGCCCAGAGTATTTGCC AACTATCTCGAGATAGTTGGC AAATACTCTGGGTTTTTG |
| NM_021196.3-1161s21c1 | TRCN0000414761 | CCGGATCGCAGGAATTGAT GAATTTCTCGAGAAATTCAT CAATTCCTGCGATTTTTTTG |
| NM_021196.3-676s21c1 | TRCN0000442365 | CCGGACCGCTCCTTAGCTGA CATTGCTCGAGCAATGTCAG CTAAGGAGCGGTTTTTTTG |
| NM_021196.2-1989s1c1 | TRCN0000038176 | CCGGCCCTATCAATATGGA CTTCAACTCGAGTTGAAGTC CATATTGATAGGGTTTTTG |

**Table S2 antibody list**

| **Target** | **Company** | **Catalogue #** | **Dilution** |
| --- | --- | --- | --- |
| NBCe-1 | Cell signalling Technology | 11867S | 1:500 |
| NBCe-2 | SigmaAldrich | HPA036621 | 1:250 |
| NBCn-1 | Abcam | ab82335 | 1:1000 |
| AE4 | Abcam | ab151133 | 1:500 |
| CAIX | Absolute antibodies | Ab00414-1.1 | 1:1000 |
| HIF-1α | BD biosciences | 610959 | 1:250 |
| HIF-2α | Novus | NB100-122 | 1:250 |
| HIF-1β (ARNT) | R&D systems | AF5630 | 1:500 |
| pMTOR | Cell signalling Technology | 2971S | 1:500 |
| MTOR | Novus biologicals | NBP2-43658 | 1:500 |
| β-actin | Santa Cruz Biotechnology | sc-1616 | 1:5000 |
| Glut-1 | Santa Cruz Biotechnology | sc-377228 | 1:1000 |
| Pdgfr-α | Abcam | Ab5460 | 1:1000 |
| Fgfr2 | Abcam | Ab10648 | 1:1000 |
| pALK | Abcam | Ab73996 | 1:1000 |
| ALK | Abcam | Ab16770 | 1:1000 |
| pcMET | Abcam | Ab5662 | 1:1000 |
| cMET | Abcam | Ab71472 | 1:1000 |
| Hgf | Abcam | Ab83760 | 1:1000 |
| VEGFr2 | Abcam | Ab39256 | 1:1000 |
| EGFr | Abcam | Ab52894 | 1:1000 |
| EGF | Abcam | Ab9695 | 1:1000 |
| ZEB1 | Abcam | Ab124512 | 1:1000 |
| ZEB2 | Santa Cruz Biotechnology | sc-271984 | 1:500 |
| E-cadherin | Santa Cruz Biotechnology | sc-71009 | 1:500 |
| Vimentin | Dako Omnis | GA63061-2 | 1:200 |
| Snail | ThermoFisher | MA5-14801 | 1:2000 |
| Slug | Santa Cruz Biotechnology | sc-166476 | 1:500 |
| Twist1 | ThermoFisher | MA5-17195 | 1:2000 |
| Twist2 | ThermoFisher | CF805832 | 1:2000 |
| GSC | ThermoFisher | PA5-40495 | 1:250 |
| WNT5a | ThermoFisher | MA5-15511 | 1:500 |
| WNT5b | ThermoFisher | PA5-72844 | 1:500 |
| P53 | Cell signalling Technology | 2524T | 1:1000 |
| Luciferase | Abcam | ab21176 | 1:1000 |
| LOX | Cell signalling Technology | 58135S | 1:1000 |
| LDHA | Cell signalling Technology | 3582S | 1:1000 |
| cMYC | Santa Cruz Biotechnology | sc-56634 | 1:500 |
| pLYC | R&D systems | MAB7500 | 1:500 |
| LYC | Cell signalling Technology | 2787T | 1:1000 |
| pLYN | Biorbyt | orb315590 | 1:1000 |
| LYN | Cell signalling Technology | 4576S | 1:1000 |

**Table S3 qRT-PCR Primer sequences**

| **Gene** | **Forward Primer Sequence** | **Reverse Primer Sequence** |
| --- | --- | --- |
| *CAI* | CCAAACATGACACCTCTCTGAA | GCCCCACATTGATAATTTCTTT |
| *CAII* | CACCCCTCCTCTTCTGGAAT | GTTAAGTTTACGGAATTTCAACACC |
| *CAIII* | CTTCGGATGATCATGGCTCT | CCAGTGAACCAAATGAAGCTC |
| *CAIV* | TGTCCAAAATAACGGGCACT | CAGTCCTCCTCCAGAAATGC |
| *CAVα* | GACATCTTGCCGGAAATAAAA | CCCGCGTAGGTCCAGTAAT |
| *CAVβ* | CATGATGGCCACTATCAGAAGA | ACAGTGCACAAGGGTTTCAA |
| *CAVI* | GTCAACAATGGCCACACAGT | ATCTCCGAGGACGCACCT |
| *CAVII* | AGAGAATTTCCCCGCAGAAG | CCCCTTCCCTCACGTACC |
| *CAIX* | CTTGGAAGAAATCGCTGAGG | TGGAAGTAGCGGCTGAAGTC |
| *CAXII* | CCCTCGAGTTCCAAGGCTA | GGGCAGGTTCAGCTTCACT |
| *CAXIII* | TTTTTGCCTGGTGTTTTGG | TTGACAGAACAATGACCCACA |
| *ACTB* | ATTGGCAATGAGCGGTTC | GGATGCCACAGGACTCCAT |
| *SLC4A4* | CCATTGCTCACATCGACAGT | CACAAGGGTTCCAGTGACTCT |
| *SLC4A5* | CAGGGAGTGATGGAGAGCTT | GAGGCTGTCCCGAGAAGA |
| *SLC4A7* | AAAGTGGAGAAATTAAAGGTAATGGA | CAACCTTGCTGAAGTCAACAGT |
| *SLC4A8* | AGAATTGAGCCACCCAAAAA | TTGGAACTCCAGGCATTTTC |
| *SLC4A9* | GGGGTCCCTTACTGAGGTGT | TAGCCCTTTCCCAGCATACA |
| *SLC4A10* | AGCCTCCCAAAAATGTTCCT | GAGCTGCTGTTCCATTTGGT |
| *Snai1* | CTTCCAGCAGCCCTACGAC | CGGTGGGGTTGAGGATCT |
| *Snai2* | TGGTTGCTTCAAGGACACAT | GCAAATGCTCTGTTGCAGTG |
| *Twist1* | AGGGCCGGAGACCTAGATT | TCATCTGGACATGTGCTTCTTC |
| *Twist2* | CATGTCCGCCTCCCACTA | GAGGGGACACTGGAGGTAGTC |
| *ZEB1* | CGAAACGCGAGGTTTTGTA | TCTAGACAGGAAATCCCACACA |
| *ZEB2* | GTTTACCGTTTATCTGAAACTAGAAGG | TTTTTGTTAGCATTTGCTTTTAGC |
| *WNT5a* | CGTCTGGAAGCAGACGTTTC | TCACGCCTCCTGATCTCC |
| *WNT5b* | TGCCTTTCCAGCGAGAAT | CCCCCTTTCCTCTTCAGGTA |
| *GSC* | CCTCCGCGAGGAGAAAGT | CGTTCTCCGACTCCTCTGAT |
| *Vimentin* | TACAGGAAGCTGCTGGAAGG | ACCAGAGGGAGTGAATCCAG |
| *LOX* | TTTCCCGCTCTCTGATTCTC | TGGAAAAGCTTGGACATGAA |
| *E-cadherin* | TGCTCTTCCAGGAACCTCTG | GCGGCATTGTAGGTGTTCA |
| *LDHA* | AACATGGCAGCCCTTTTATG | ATTGGAGTTTGCAGTGACCA |
| *Glut1* | GCCCATGTATGTGGGTGAA | AGTCCAGGCCGAACACCT |
